# A Novel Method for Fabricating Wearable, Piezoresistive, and Pressure Sensors Based on Modified-Graphite/Polyurethane Composite Films

**DOI:** 10.3390/ma10070684

**Published:** 2017-06-22

**Authors:** Yin He, Wei Li, Guilin Yang, Hao Liu, Junyu Lu, Tongtong Zheng, Xiaojiu Li

**Affiliations:** 1 School of Textiles, Tianjin Polytechnic University, Tianjin 300387, China; smileheyin@yeah.net (Y.H.); lweiyuanb@163.com (W.L.); yangglin@163.com (G.Y.); lujunyu0301@163.com (J.L.); 13920038537@139.com (T.Z.); 2 Institute of Smart Wearable Electronic Textiles, Tianjin Polytechnic University, Tianjin 300387, China; lixiaojiu@tjpu.edu.cn; 3 School of Art and Fashion, Tianjin Polytechnic University, Tianjin 300387, China; 4 Key Laboratory of Advanced Textile Composite Materials, Ministry of Education of China, Tianjin 300387, China

**Keywords:** modified graphite, polyurethane composite film, mechanical properties, electrical conductivity, piezoresistive sensor

## Abstract

A wearable, low-cost, highly repeatable piezoresistive sensor was fabricated by the synthesis of modified-graphite and polyurethane (PU) composites and polydimethylsiloxane (PDMS). Graphite sheets functionalized by using a silane coupling agent (KH550) were distributed in PU/*N*,*N*-dimethylformamide (DMF) solution, which were then molded to modified-graphite/PU (MG/PU) composite films. Experimental results show that with increasing modified-graphite content, the tensile strength of the MG/PU films first increased and then decreased, and the elongation at break of the composite films showed a decreasing trend. The electrical conductivity of the composite films can be influenced by filler modification and concentration, and the percolation threshold of MG/PU was 28.03 wt %. Under liner uniaxial compression, the 30 wt % MG/PU composite films exhibited 0.274 kPa^−1^ piezoresistive sensitivity within the range of low pressure, and possessed better stability and hysteresis. The flexible MG/PU composite piezoresistive sensors have great potential for body motion, wearable devices for human healthcare, and garment pressure testing.

## 1. Introduction

Piezoresistive sensors which transduce stress imposed on the sensor into a resistance signal have been applied in extensive fields such as sensitive e-skin, wearable health care devices, and human motion detection [1,2,3,4,5]. The conducting polymeric composites are promising materials for such sensors because of their flexibility and facile fabrication. Conductive fillers are dispersed evenly into the polymer matrix of composites and form an electrical network; therefore, the electrical resistance of the composites can be changed by mechanical deformation, which is defined as a sensing mechanism [6,7].

Rubber-like materials, which have low modulus, high elasticity, and good flexibility, are commonly used as a substrate matrix for supporting and fixing conductive fillers in composites such as nitrile-butadiene rubber (NBR) [8,9,10], silicone rubber (SR) [11], styrene-butadiene rubber (SBR) [12,13,14], polydimethylsiloxane (PDMS) [15], and segmented polyurethane (PU) [16,17]. Zhang et al. [18] have researched the highly stretchable, sensitive, and flexible sensors based on silver nanoparticles and carbon nanotube composites with PDMS substrates. Yao et al. [19] reported that a piezoresistive sensor was fabricated with graphene and a polyurethane sponge, which enhances the pressure sensitivity in order to reach a desired magnitude for artificial electronic skin applications. According to the previous studies, carbon materials have been researched as main filler components to improve the electrical and mechanical properties of polymer composites, such as carbon black [20], graphite [21,22], carbon nanotubes [23,24], and graphene [25]. However, carbon nanotubes and graphene have limited large-scale applications due to the high cost [26,27,28]. Alternatively, graphite, as a good conductor and inexpensive and abundant material, is being used to increase the electrical conductivity of polymeric matrices [29,30]. Chen et al. [11,21] fabricated SR/graphite nanosheet composite films with finger pressure sensitivity (0.3–0.7 Mpa). The electrically conductive NBR nanocomposites with different concentrations of graphite were investigated by Al-solamy et al. [9]. The electrical conductivity of the NBR/graphite nanoplates was changed by more than five orders of magnitude under the condition of 60% compression and by more than two orders of magnitude under 6 MPa pressure. The composite was found to be most sensitive to compressive strain in the range of the percolation transition.

Although conductive polymer composites are used as the sensing materials for piezoresistive sensors, they still face some challenges [19]. Surface modification of conductive fillers was an effective approach for improving the sensitivity and reliability of the sensors, because the conductive filler particles can be evenly dispersed into the polymeric matrix. Wang et al. [30] fabricated the graphite nanosheet/polystyrene composites to successfully improve electrical and mechanical properties by applying a supercritical water treatment to the graphite nanosheets. Chung et al. [31] have discussed that by modifying a phenol or 4-phenylethanol group, graphite was linked with polyurethane. The electric conductivity of the composites was improved sharply with the increase of the graphite content.

In this work, we presented a facile method for fabricating a flexible, low-cost, piezoresistive pressure sensor with high sensitivity and reliability by using a silane coupling agent to link graphite fillers with the PU elastomer. The graphite sheets modified by using the silane coupling agent (KH550) were distributed into PU/*N*,*N*-dimethylformamide (DMF) solution, and then the mixed solution was molded into modified-graphite/PU (MG/PU) composite films. Subsequently, we investigated the mechanics and electrical properties of the composite films, and the properties (linearity, hysteresis, and repeatability) of the MG/PU piezoresistive sensor. The MG/PU piezoresistive sensors could be used in a wide variety of sensing applications including human motion sensing, pressure detection, and tactile sensors for artificial skin.

## 2. Materials and Methods

### 2.1. Materials

Natural graphite powder (particle size <30 µm and purity >99.9%) were purchased from Kermel Chemical Reagent Co., Ltd. (Tianjin, China). Polyurethane resin (PU, solid content: 30%, viscosity: 160–220 Pa/s, 100% modulus: 2 MPa, tensile strength: 50 MPa, elongation: 800%) was supplied by HuaDa Industrial Co., Ltd. (Yantai, China). The main raw materials of PU contain 4,4′-diphenylmethane diisocyanate (MDI), poly(ethylene propylene adipate)glycol (PEPA), 1,4-butylene glycol(BDO), and *N*,*N*-dimethylformamide (DMF). Neat PU was synthesized by the method of solution polymerization which uses DMF as a solvent to combine with PEPA:BDO:MDI with a molar ratio = 1:3.1:4.2 at 75 °C. A linear molecular construction of PU consists of soft segments (SS) based on polyol polyester and hard segments (HS) formed by MDI and BDO (chain extender). *N*,*N*-dimethylformamide (DMF) and anhydrous ethanol, used as solvents for preparing the graphite and the composites, were purchased from Tianjin Kemiou Chemical Reagent Co., Ltd. (Tianjin, China). Silane coupling agent γ-aminopropyl-triethoxy silane (KH550) was supplied by Beijing Chemical Reagent Company (Beijing, China) and was used to modify the surface activities of graphite.

### 2.2. Surface Modification of Graphite

Pristine graphite powder was added into the modified solution which was prepared according to the proportion of absolute ethyl alcohol:distilled water:silane coupling agent (KH550, Beijing Chemical Reagent Company, Beijing, China) = 9:1:2.5. The mixed suspension was ultrasonically dispersed for 30 min. The well-dispersed solution was stirred using a magnetic stirrer at 60 °C for 4 h, and was then repeatedly washed by using deionized water with a high speed desk centrifuge (TGL20M Beijing Huarui Scientific Equipment Co., Ltd, Beijing, China) three times, which lasted for at least 15 min each time. The modified graphite was dried in a vacuum oven for 12 h.

### 2.3. Fabrication of Composite Films

The composite samples were prepared by the solution compounding method. Different weight fractions of modified graphite between 0 and 50 wt %, as shown in Table 1, were introduced in 20 mL DMF solvent. The solution was stirred evenly and ultrasonic dispersion was performed for 30 min. A certain amount of PU resin was added into the blend under mechanical stirring with a high-speed mixer for 10 min. After mixing was completed, the fillers were evenly dispersed in the polymer matrix. The suspensions were standing in vacuum for 12 h to avoid the trapped bubbles from influencing the intensity of composite films. MG/PUcomposite films were processed into samples with the size of 4 × 7 cm^2^ and the thickness of 100 μm by rod coating on glass molds and were dried in vacuum oven for 3 h. The specific sizes of the composites’ thickness can be seen in Table 2.

### 2.4. Testing Properties

#### 2.4.1. Particle Size Analysis

A Delza Nano Particle size analyzer (Beckman Counlter, Brea, CA, USA) was used to measure the diameter of the graphite sheets. The suspensions were prepared by adding pristine graphite and modified graphite into DMF, respectively, and were ultrasonically dispersed for 30 min before analysis. After the settlement of the suspensions during the last 12 h, those diameters were measured again.

#### 2.4.2. Fourier Transform Infrared Spectroscopy

Fourier transform infrared (FTIR) spectra of the MG/PU composite films were acquired by a BRUKER TENSOR 27 (Bruker Corporation, Karlsruhe, Germany), and the scanning frequency range was 4000–400 cm^−1^.

#### 2.4.3. Morphological Analysis

The HITACHI S-4800 field-emission scanning electron microscope (FE-SEM) (Hitachi, Ltd., Tokyo, Japan) was used to observe the morphology of the modified-graphite/PU composite films. Small pieces of the samples were placed in the sample holder to study the surface.

#### 2.4.4. Mechanical Properties Analysis

Tensile mechanical tests were carried out on a Universal testing machine (YG065, Laizhou Electronic Instrument Co., Ltd., Yantai, China) with a 500.0 N load cell (±0.5% at 1/200 load cell capacity). The MG/PU composites samples were prepared into 20 mm wide, 70 mm long strips with a gauge length of 50 mm, and loaded in tension with a crosshead rate of 200 mm/min.

#### 2.4.5. Electrical Characterization

Electrical conductivity experiments were conducted by Broadband Dielectric Spectroscopy (BOS50, NOVO Control GmbH Co., Montabaur, Germany). The dielectric permittivity of pristine and MG/PU composite films was measured in the frequency range from 10^−1^ Hz to 10^7^ Hz by using 20 mm diameter gold disk electrodes. The testing temperature was the adjusted room temperature (20 °C).

#### 2.4.6. Pressure-Sensing Behavior of the Composite Films

**Figure 1:**
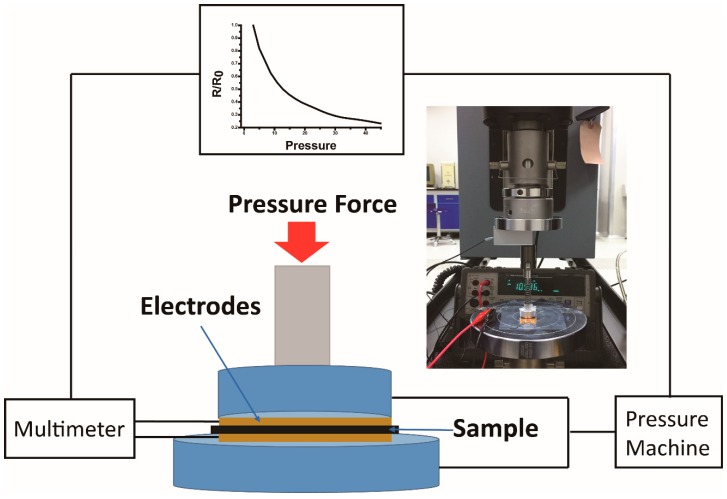
Experimental set up for measurements of electrical resistance of the modified-graphite/polyurethane (MG/PU) composite films as a function of dynamic pressure and compressive strain.

The pressure-sensing test was performed on circular samples. Figure 1 shows the schematic diagram of this measurement. The diameter of the composites samples was 10 mm with the thickness of about 100 μm, and the specific thickness parameters of the samples can be seen in Table 2. Two copper sheets were applied on both sides of the films to ensure good contact with the instruments. The samples were set into the Universal testing machine (INSTRON 5969, Norwood, MA, USA) and loaded with a constant compressive force at the speed of 10 mm/min with a 500.0 N load cell (±0.5% at 1/200 load cell capacity) to measure the pressure. Meanwhile, the resistance of the samples was monitored by a Digital Dual Display Multimeter (U3402A, Agilent Technologies, Santa Clara, CA, USA). The real-time compressive stress-strain and resistance of the samples were recorded by a computer and LabVIEW software. Multi-cycles of compressive force loading and unloading were also conducted to further study the dynamic sensing behavior with increasing the loading speed to 20 mm/min.

## 3. Results and Discussion

**Figure 2:**
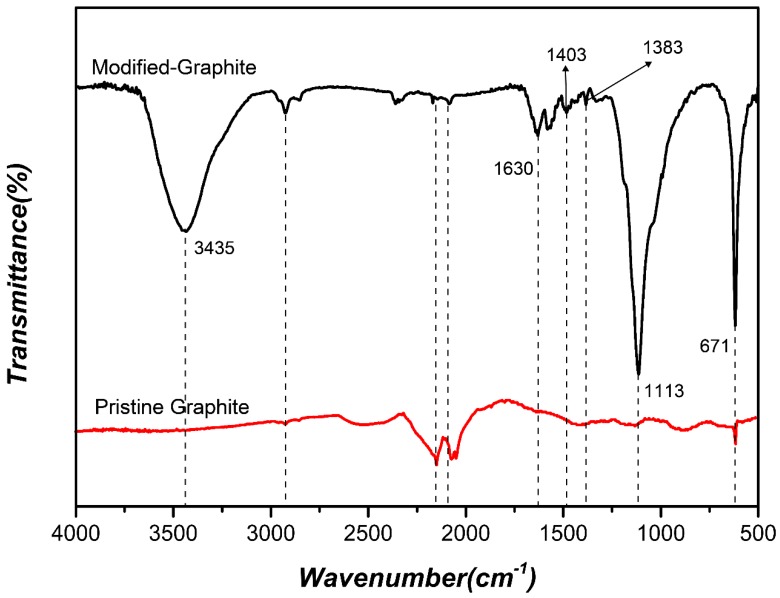
Fourier Transform Infrared (FTIR) spectra of pristine graphite and modified graphite.

Compared with the FTIR spectrum of natural graphite, the spectrum of modified graphite in Figure 2 shows that a new –OH stretching peak appears at 3435 cm^−1^, and the peaks at 671 cm^−1^ represent the stretching vibrations of C=O. The peaks at 1630 cm^−1^ and 1113 cm^−1^ correspond to N–H and Si–O. Some absorption peaks at 1403 cm^−1^ and 1383 cm^−1^ also appear on the FTIR of modified graphite. The results indicate that hydrolysate silanol of the silane coupling agent has been grafted successfully onto the surface of the graphite sheets. Hydrolysis of the alkoxide groups of KH550 produces silanol (SiOH) groups, which can combine with the oxygen-containing groups of graphite sheets by physical absorption and/or chemical bonds [31].

Table 3 shows the diameters of pristine graphite and the modified graphite sheets. The diameter of modified graphite reaches 3683.5 nm and is approximate 16% of that of the untreated graphite sheets. After 12 h sedimentation, the diameters of the pristine graphite and modified graphite are, respectively, 163% and 82% more than those of the graphite sheets before solution sedimentation. These results illustrate that large space obstacle exists between graphite sheets because of KH550, which can reduce the aggregation of graphite sheets. Thus, modified graphite can be dispersed easily into the solvent which has a positive effect on the homogeneity and stable distribution of the graphite in the polymers.

**Figure 3:**
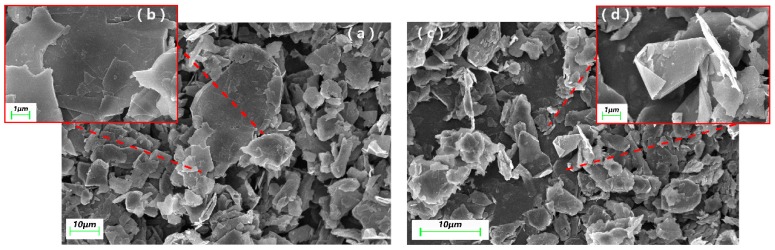
Scanning electron microscope (SEM) images of pristine graphite (**a**) with magnification at 1 μm (**b**) and modified graphite (**c**) with magnification at 1 μm (**d**).

Figure 3 shows the scanning electron microscope (SEM) images of the unmodified and modified graphite sheets. As we can see in Figure 3a,b, most of the natural graphite are flakes, and some are particles with multi-layers. The size of the graphite flakes is not uniform, but the average value of the diameter is about 20 μm. It is observed in Figure 3c that the modified graphite powder has basically a random sheets structure, and the diameter of the sheets is <10 μm. There is significant peeling between the graphite layers, and the thickness of the sheets is about 500 nm as shown in Figure 3d.

**Figure 4:**
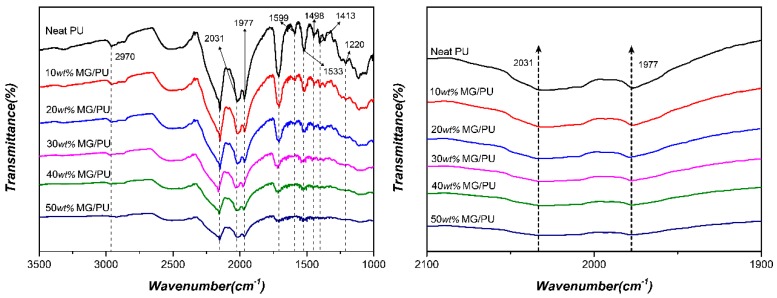
**Left:** FTIR spectra of MG/PU composite films with different weight fractions of graphite fillers. **Right**: Spectra scaled in the absorbance region of the carbonyl groups.

Figure 4 shows the FTIR spectra of the MG/PU composites with different amounts of the fillers. The FTIR spectrum of the unfilled PU exhibits the typical bands for polyurethanes, which are the stretching vibrations of N–H at 2970 cm^−1^, the aromatic C–C ring stretch peak at 1599 cm^−1^ and 1498 cm^−1^, the C–H bending peak at 1533 cm^−1^, the –OH bending peak at 1413 cm^−1^, and the C–O carbonate group stretching band at 1220 cm^−1^. However, the obvious decrease for the N–H bands implies that the group in KH550 would form a hydrogen bond with the oxygen in PU, thereby forming a bridge of the fillers and matrix. Furthermore, the stretching peak of the C=O groups in the hard segments of PU associated with the non-hydrogen bonded carbonyl group and the stretching peak of the C=O groups with the bonded carbonyl group at 2031 cm^−1^ and 1977 cm^−1^ are observed, respectively. Compared with these bands, the significant decrease of the peak at 2031 cm^−1^ is due to the interfacial interaction between the C=O groups of the hard segments of PU and the hydroxyl groups in the modified graphite layers by hydrogen bonds. Thus, the carboxyl groups contribute to grafting the graphite fillers and PU matrix.

**Figure 5:**
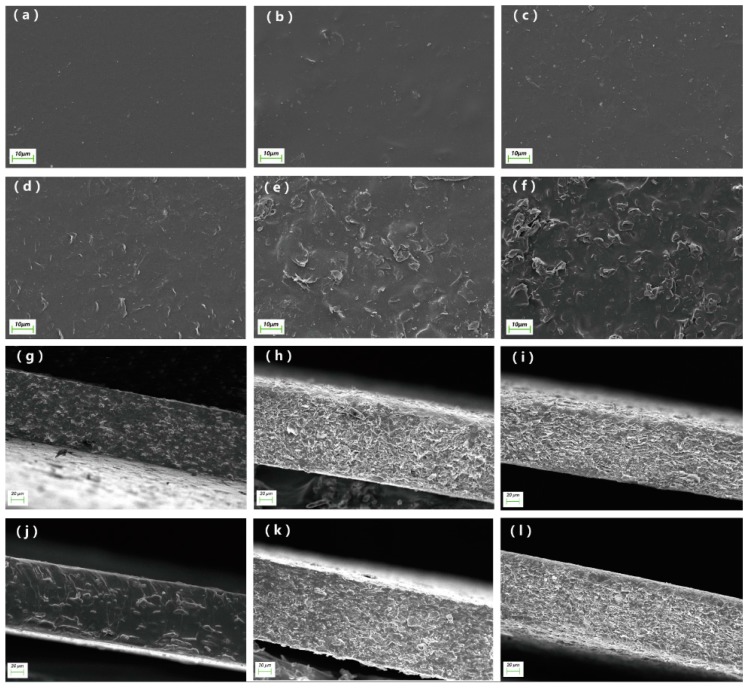
SEM images of the surfaces for the neat PU and MG/PU composites with various modified graphite contents (**a**–**f**): (**a**) Neat PU; (**b**) 10 wt % MG/PU; (**c**) 20 wt % MG/PU; (**d**) 30 wt % MG/PU; (**e**) 40 wt % MG/PU; (**f**) 50 wt % MG/PU. The cross-sectional SEM images of the pristine graphite/PU(G/PU) and MG/PU composites with various fillers contents (**g**–**l**): (**g**) 10 wt % G/PU; (**h**) 30 wt % G/PU; (**i**) 50 wt % G/PU; (**j**) 10 wt % MG/PU; (**k**) 30 wt % MG/PU; (**l**) 50 wt % MG/PU.

Figure 5a shows that the surface of neat PU is smooth. In the case of the MG/PU composites, much rougher surfaces are seen by adding modified-graphite into the PU matrix, as shown in Figure 5b–f. Although the degree of surface roughness increases with the rise of the modified-graphite content in the composites, well dispersed modified-graphite sheets are observed for the composite with a modified graphite content of 30 wt %, as seen in Figure 5d. However, when the mass content of the modified graphite is up to 40–50 wt %, the overlapping graphite sheets appear obviously on the composite surfaces, as seen in Figure 5e–f, which demonstrate that the modified graphite sheets are closely connected due to its high content, and its compatibility with the PU becomes poor and therefore aggregation occurs. The same phenomenon can be obtained from the SEM cross-section images, as shown in Figure 5g–l. Figure 5g–i shows that with increasing graphite content, we can see that more overlapping graphite sheets appear in pristine graphite/PU (G/PU). However the better dispersibility and compatibility of the modified graphite in polyurethane can be seen in Figure 5j–l.

**Figure 6:**
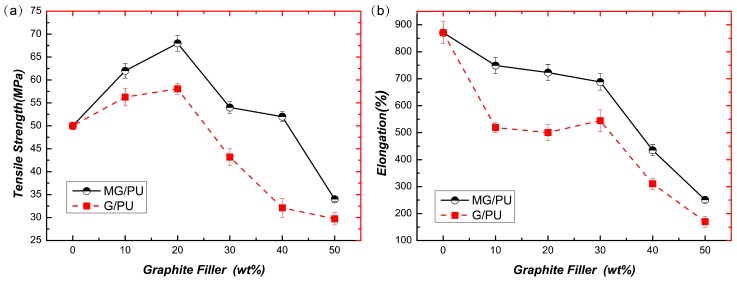
The curves of filler content vs. tensile strength (**a**) and elongation at break (**b**) for the MG/PU composites and G/PU composites.

Figure 6a shows that with the increasing graphite content, the tensile strength of the composite films first increases and then decreases. When the content of the graphite filler is 20 wt %, the stress reaches its peak at 68 ± 1.7 MPa of MG/PU and 58.07 ± 1.5 MPa of G/PU, which demonstrates that the addition of the modified graphite filler has a reinforcement function for the composites [28]. When the graphite content increases continuously, the tensile stress exhibits a decreasing tendency. As shown in Figure 6b, with the increasing graphite filler, the elongation at break of the composite films shows a decreasing tendency, approximately from 870% to 250% of MG/PU and to 170% of G/PU. The composite films with 20 wt % modified graphite have an increased tensile strength of 36% and less than 17% loss of elongation at breaking compared with the pure PU film. In addition, compared with the mechanical properties of graphite derivative/PU composites that include fabrication by other methods [28,32], the tensile strength and elongation at break of the 20 wt % MG/PU composites were significantly improved in the present work.

**Figure 7:**
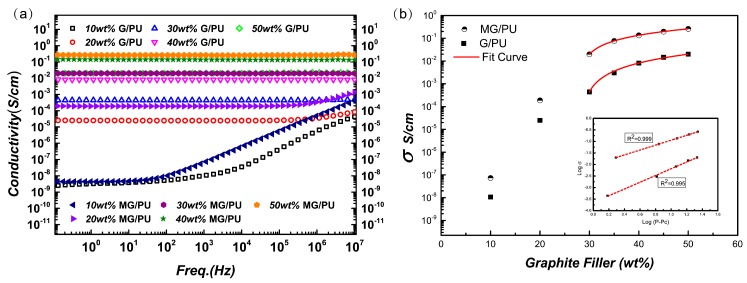
(**a**) Frequency dependence of the conductivity at room temperature for G/PU and MG/PU films with different filler weight fractions (wt %); (**b**) Dependence of the conductivity on the modified graphite weight fraction P. The curves are the fits to the percolation theory.

In order to study the electrical properties of the MG/PU composites, the dielectric permittivity and conductivity effect on concentration, frequency, and percolation threshold have been investigated. Figure 7a shows that the real parts of the complex conductivity vary with the frequency domain for the composites with pristine graphite and modified graphite by measuring at fixed temperatures (20 °C).

The complex dielectric permittivity of the samples can be expressed by Equation (1):

ε*(ω) = ε′(ω) − iε″(ω),(1)

The complex electrical conductivity of the samples can be expressed by Equation (2):

σ*(ω) = σ′(ω) + iσ″(ω)(2)

And Equations (1) and (2) are related by:

σ*(ω) = iωε_0_ε*(ω) (3)

where ε_0_ is the vacuum permittivity. Therefore,

σ′(ω) = ωε_0_ε″(ω) and σ″(ω) = ωε_0_ε′(ω) (4)

As can be seen, the conductivity of the composites greatly increases in magnitude with increasing filler content and frequency. For the composites with a filler content less than 30 wt %, the σ′(ω) of Figure 7a shows linear plots in the high frequency region which reached 10^−4^ S/cm and 10^−3^ S/cm for 10 wt % and 20 wt % MG/PU, respectively. However, for the composites with a filler content more than or equal to 30 wt %, the conductivity (≥10^−2^ S/cm) is subtly influenced by the changed frequency. The electrical conductivity of 50 wt % MG/PU was 0.26 S/cm, which is one order of magnitude higher than that of the 50 wt % G/PU. It also can be observed that the conductivity value of the composites which are filled with the modified graphite sheets is higher than that of the G/PU composites at the same frequencies.

The frequency-independent conductivity is associated with the highly interconnected electrical network with a high graphite content. Thus, it can be suggested that the critical filler content should be around 30 wt % for the composites, which can be described by a scaling law according to the classical percolation theory [33,34]:

σ = σ_0_(*P* − *Pc*)*^t^* for *P > Pc* (5)

where σ_0_ is a constant, *Pc* is the weight fraction of the filler or percolation threshold, σ is the conductivity of the composite, *P* is the filler content, and *t* is the critical scaling exponent. The critical exponent *t* is related to the dimensionality of the conductive network. The data are fitted to the scaling law and evaluated by multiple non-linear regression analysis in Figure 7b. For the Graphite/PU composite, the percolation threshold and exponent *t* are calculated by using Equation (5), which are 28.46 wt % and 1.45, respectively. For the MG/PU composites, the above values are 28.03 wt % and 1.07, respectively. These values are slightly lower than those reported for other composites containing graphite [34]. Moreover, the *t* values obtained for the modified-graphite filler is lower than the universal value for two-dimensional percolating systems (*t* = 1.3). That could be considered as the reduction of the dimensionality in the conductive network by adding modified-graphite filler with KH550. Both the unmodified and modified graphite effectively increased the electrical conductivity. However, the difference of the percolation threshold is minor for the two kinds of graphite filler, in comparison with the electrical properties of graphite polymeric materials in reported works [16,26,31,35].

**Figure 8:**
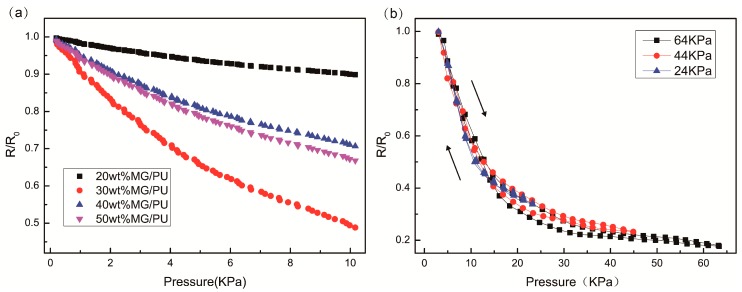
(**a**) The resistivity change of MG/PU composite films with applied pressure; (**b**) Hysteresis curves of the 30 wt % MG/PU composite films under different loading-unloading pressure cycles.

The force-sensing capability of the MG/PU composites is shown in Figure 8a, and the resistivity changes of all of the composites generally decrease logarithmically by imposing a uniform force in the range of 0–10 KPa. By applying an external pressure on the composites, the compressive deformation could enhance the contact among the inner conducting particles in the matrix, resulting in more electrical paths in the conductive network and resistance variation. To quantify the relationship of the variation of resistivity and mechanical deformation, the sensitivity can be expressed as [36]:

S = δ (∆R/R_0_)/δP (6)

∆R = R − R_0_ (7)

where P is the relative applied pressure, and R and R_0_ are the resistance and initial resistance, respectively. The numerical values of the composites’ sensing response to different pressures can be found in Table 4. All composites exhibit a higher response in the low pressure range. For 20 wt % MG/PU composites, the sensitivity is 0.047 KPa^−1^ with the load of 0.2 KPa. The sensitivity of 30 wt % MG/PU composites is found to be about 0.274 KPa^−1^ for the region corresponding to a pressure smaller than 0.2 KPa. Meanwhile, the sensitivity of 40 wt % and 50 wt % MG/PU exhibits 0.149 KPa^−1^ and 0.163 KPa^−1^ respectively within the range of low pressure (<0.2 KPa). Moreover, the change of the filler content causes the variation of the slope in the resistivity-pressure curve. As shown in Table 4, the MG/PU composite with 30 wt % filler content possesses the highest piezoresistive sensitivities at 0–0.2 KPa pressure, which is equal to that reported by Yao et al. [19] in their work using graphene-polyurethane sponge as a pressure sensor with the sensitivity of 0.26 KPa^−1^. These results may be explained by the fact that near the percolation threshold the electrical resistance of the composites with applied stress reveals the largest variation [18]. The sensitivity of 30 wt % MG/PU composites is higher than that of the silver nanowire composites (0.08 kPa^−1^) reported by Quan et al. [37], but similar to that of other carbon nanotube composites when compared with previously reported pressure sensors [38].

The difference in the relative resistivity change ratio between the pressing and releasing process is also a significant factor for evaluating the performance of the force-sensing capability. Figure 8b illustrates the resistance-stress curves for 30 wt % MG/PU films under different compressive stresses. For large deformation, a decrease of the number of conducting contacts and destruction of the conductive network should be considered [26]. We can see a particularly sharp tendency of the resistivity change in the low pressure region, and then the speed of decrease slows to a constant value under the condition of continuous applied pressure.

Moreover, there is a small hysteresis in the response of the 30 wt% MG/PU which is the composites around the percolation threshold. The hysteresis error of the curves under a compress/release cycle at 64 KPa, 40 KPa, and 20 KPa is ±5%, ±4%, and ±0.54%, respectively, according to Figure 8b. The figure illustrates that the sensing curves of the 30 wt % MG/PU composites has no hysteresis under the low range of pressure. It is because fillers are dispersed in stable state throughout the PU matrix. The recovery property will make the detection more accurate and reliable [39].

**Figure 9:**
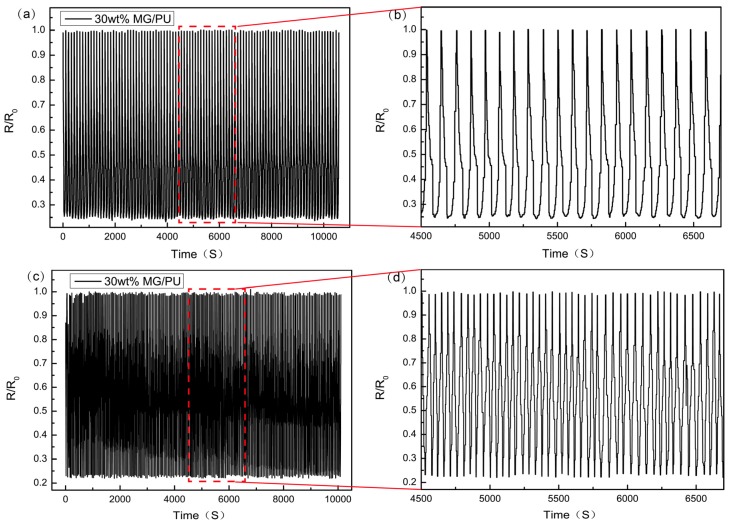
(**a**,**c**) The dynamic resistivity variation of 30 wt % MG/PU film under cyclic loading/unloading with different speed rates of 10 mm/min (**a**), 20 mm/min (**b**) and the pressure of 64 KPa. (**b**,**d**) Enlarged views of the selected area in Figure 9a,c respectively.

Furthermore, the piezoresistive stability of the 30 wt % MG/PU composites was tested by automatic pressing and releasing at different loading speeds for more than 10,000 s. For the dynamic force loading-unloading, it is observed in Figure 9 that the resistivity decreases with increasing pressure and increases with decreasing pressure in every cycle. By increasing the loading speed to 20 mm/min, the curve change is accelerated. However, the range of the change rate is from 1.0 to 0.2, which is the same as the range at 10 mm/min. Therefore, the 30 wt % MG/PU composite shows great responsiveness and resistance retention capacity to repeating pressure that almost remains unchanged as a whole. The phenomenon is thought to be attributed to the more homogeneous dispersion of fillers that construct the stable conductive network.

**Figure 10:**
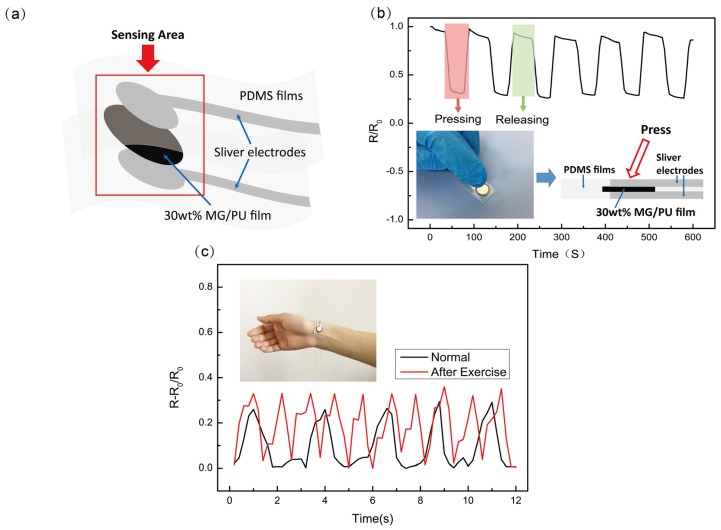
Detection of the resistance responses to dynamic loading and unloading cycles. (**a**) Schematic of the flexible piezoresistive sensor; (**b**) pressing; (**c**) piezoresistive response of the sensor wrapped around the wrist before and after exercise.

To demonstrate the applicability of the wearable piezoresistive sensor (which was obtained by constructing silver electrodes on both sides of the 30 wt % MG/PU composites, and which were sealed by PDMS films, as shown in Figure 10a), it can simultaneously be used to detect the pressing and bending forces. Figure 10b shows that a rapid decrease in resistance is clearly observed when the finger presses the sensing area, and the resistance returns to its original state after the finger releasing.

Besides motion monitoring, this composite sensor can be applied for the detection of subtle physiological signals such as the pulse wave of the human body, as shown in Figure 10c. The resistive signal of the sensor before and after exercise is recorded with a repeatable pulse frequency of 63 beats per min and 120 beats per min, respectively. The pressure sensor can clearly distinguish the signal of the normal pulse from the pulse after exercise. These results can be used to explore the sensor’s ability to detect human motion and its potential use in healthcare monitoring or e-skins.

## 4. Conclusions

In summary, a flexible, low-cost, and highly repeatable piezoresistive pressure sensor was developed based on modified graphite and PU. The FTIR spectrum, particle size analysis, and SEM results indicate that the hydrolysate silanol of KH550 has been coated successfully on the surface of the graphite sheets whose diameters can reach about 3–4 μm and are approximately 16% of that of the pristine graphite. The modified-graphite sheets can be well dispersed into the PU matrix to improve the interaction of the fillers and the polymer. By continuously adding modified graphite, the tensile strength of the MG/PU films is first enhanced and is then reduced, while the elongation generally decreases. The tensile strength and elongation at breaking of the MG/PU composites is stronger when compared with that of the G/PU films with any corresponding filler concentration. The electrical conductivity of the composite films can be influenced by filler modification and concentration, and the percolation threshold of MG/PU was 28.03 wt %. Compared with that of G/PU (28.46 wt %), the improvement of electrical conductivity by using KH550 for the modification of graphite is not obvious. Under liner uniaxial compression, the 30 wt % MG/PU composite films exhibited 0.274 kPa^−1^ piezoresistive sensitivity, and possessed better stability and hysteresis within the small pressure region. For the uniaxial compression test, with both static or dynamic force, the piezoresistive effect has been enhanced by increasing the filler content for the composites, and has shown good sensitivity, stability, and recovery performance of the electro-mechanical consistency near the percolation threshold of ca. 30 wt %. The sensing capacity of the MG/PU composites is sensitive and repeatable as compared with previously reported sensors, which were assembled with high-cost nanomaterials and sophisticated fabrication methods. Therefore, the flexible MG/PU composite piezoresistive pressure sensors have great potential in wearable devices for pressure testing, human body motion, healthcare detection, and garments and robotics applications.

## Figures and Tables

**Table 1:** Composition of modified-graphite/polyurethane (MG/PU) composite films.

Sample Name	Polyurethane (g)	Modified Graphite (g)	Filler Content by Weight (wt %)
**Pure PU**	4.8	–	0
**10 wt % MG/PU**	4.8	0.53	10
**20 wt % MG/PU**	4.8	1.2	20
**30 wt % MG/PU**	4.8	2.1	30
**40 wt % MG/PU**	4.8	3.2	40
**50 wt % MG/PU**	4.8	4.8	50

**Table 2:** The thickness of the modified-graphite/PU composite films.

Modified Graphite Content (wt %)	0	10	20	30	40	50
**Thickness (mm)**	0.096	0.105	0.110	0.128	0.135	0.151
**Coefficient of Variation (%)**	0.10	0.11	0.06	0.06	0.06	0.09

**Table 3:** Average Diameters of Graphite Sheets (nm).

Experiments	Before Settlement (a)				After 12 h Settlement (b)			
	Graphite		Modified Graphite		Graphite		Modified Graphite	
	Diameter	Std. Dev.	Diameter	Std. Dev.	Diameter	Std. Dev.	Diameter	Std. Dev.
**1st**	21,203	899.1	3443.5	27.8	53,500	1896.6	6924.3	122.3
**2nd**	19,422	881.9	3924.3	37.5	53,274	1902.7	6543.7	97.9
**Mean Size**	**20,312.5**	**890.5**	**3683.5**	**32.65**	**53,387**	**1899.65**	**6734**	**110.1**

**Table 4:** Piezoresistive sensitivities of MG/PU composites (KPa^−1^).

Types of MG/PU Composites	Corresponding Pressure			
	0–0.2 KPa	0.2–1 KPa	1–5 KPa	5–10 KPa
**20 wt % MG/PU**	0.047	0.016	0.012	0.007
**30 wt % MG/PU**	0.274	0.091	0.063	0.031
**40 wt % MG/PU**	0.149	0.050	0.035	0.019
**50 wt % MG/PU**	0.163	0.054	0.039	0.022
